# Clinical and molecular characterization of a cohort of patients with novel nucleotide alterations of the Dystrophin gene detected by direct sequencing

**DOI:** 10.1186/1471-2350-12-37

**Published:** 2011-03-11

**Authors:** Francesca Magri, Roberto Del Bo, Maria G D'Angelo, Alessandra Govoni, Serena Ghezzi, Sandra Gandossini, Monica Sciacco, Patrizia Ciscato, Andreina Bordoni, Silvana Tedeschi, Francesco Fortunato, Valeria Lucchini, Matteo Cereda, Stefania Corti, Maurizio Moggio, Nereo Bresolin, Giacomo P Comi

**Affiliations:** 1 Dino Ferrari Centre, Department of Neurological Sciences, University of Milan, I.R.C.C.S. Foundation Cà Granda, Ospedale Maggiore Policlinico, Milan, Italy; 2 Scientific Institute IRCCS E. Medea, Bosisio Parini, Lecco, Italy; 3 Laboratory of Medical Genetics, I.R.C.C.S. Foundation Cà Granda, Ospedale Maggiore Policlinico, Milan, Italy

## Abstract

**Background:**

Duchenne and Becker Muscular dystrophies (DMD/BMD) are allelic disorders caused by mutations in the dystrophin gene, which encodes a sarcolemmal protein responsible for muscle integrity. Deletions and duplications account for approximately 75% of mutations in DMD and 85% in BMD. The implementation of techniques allowing complete gene sequencing has focused attention on small point mutations and other mechanisms underlying complex rearrangements.

**Methods:**

We selected 47 patients (41 families; 35 DMD, 6 BMD) without deletions and duplications in *DMD *gene (excluded by multiplex ligation-dependent probe amplification and multiplex polymerase chain reaction analysis). This cohort was investigated by systematic direct sequence analysis to study sequence variation. We focused our attention on rare mutational events which were further studied through transcript analysis.

**Results:**

We identified 40 different nucleotide alterations in DMD gene and their clinical correlates; altogether, 16 mutations were novel. DMD probands carried 9 microinsertions/microdeletions, 19 nonsense mutations, and 7 splice-site mutations. BMD patients carried 2 nonsense mutations, 2 splice-site mutations, 1 missense substitution, and 1 single base insertion. The most frequent stop codon was TGA (n = 10 patients), followed by TAG (n = 7) and TAA (n = 4). We also analyzed the molecular mechanisms of five rare mutational events. They are two frame-shifting mutations in the *DMD *gene 3'end in BMD and three novel splicing defects: IVS42: c.6118-3C>A, which causes a leaky splice-site; c.9560A>G, which determines a cryptic splice-site activation and c.9564-426 T>G, which creates pseudoexon retention within IVS65.

**Conclusion:**

The analysis of our patients' sample, carrying point mutations or complex rearrangements in *DMD *gene, contributes to the knowledge on phenotypic correlations in dystrophinopatic patients and can provide a better understanding of pre-mRNA maturation defects and dystrophin functional domains. These data can have a prognostic relevance and can be useful in directing new therapeutic approaches, which rely on a precise definition of the genetic defects as well as their molecular consequences.

## Background

Dystrophinopathies are a heterogeneous group of disorders caused by mutations in dystrophin (*DMD*) gene [1]. Duchenne Muscular Dystrophy (DMD; OMIM #310200) is the most severe form, with an incidence of 1 in 3,500 newborn males [2] and loss of independent ambulation before the age of 14 years [3]. Becker Muscular Dystrophy (BMD; OMIM #300376) is a milder and more heterogeneous allelic disorder with near-normal life expectancy [4].

Until recently, the genetic approach was limited to methods that were able to detect large deletions and duplications because the large size of the *DMD *gene made finer-scale mutation analysis prohibitively time consuming. In DMD patients, they account for 65% and 10% of mutations, respectively; in BMD, the mutation detection rate is 85% [5].

The recent implementation of diagnostic techniques with possibility to perform high-throughput direct sequencing of the *DMD *gene [6] has increased diagnostic sensitivity and focused attention on small point mutations and more complex gene rearrangements that otherwise would hardly be detected.

The analysis of both types of mutations, the study of their positions along the *DMD *gene and their effects on the transcript processing and protein levels helps to define the correlations with clinical presentation and to build a clear understanding of the molecular pathology of dystrophinopathies. The described single nucleotide substitutions and small nucleotide changes along the coding sequence, together with other more complex *DMD *gene rearrangements, are listed on the Leiden website http://www.dmd.nl/ and described in several works [7-12]. Knowledge about these small nucleotide changes can provide a better understanding of dystrophin functional domains, as well as the mechanisms underlying transcriptional and post-transcriptional events.

The overall number of described pre-RNA maturation defects and deep intronic mutations in the DMD gene is still relatively small, and their effects on mRNA processing and protein expression are often unknown [10,13-17]. The clinical usefulness of drawing conclusions about the prognosis of a given mutation based on a single or few reports is questionable.

Furthermore, the development of several new therapeutic approaches for DMD strongly relies on a precise definition of the genetic defects as well as their molecular consequences. Indeed, fundamental areas of therapeutic research on DMD/BMD are currently based on the usage of molecules that promote the read-through of pathological stop codons created by point mutations [18,19]. A similar need for deeper understanding applies to the entire field of exon-skipping technologies based on our current knowledge of the process, which naturally regulates dystrophin mRNA maturation [20]. From this point of view, the analysis of naturally occurring aberrant splicing events might be instructive regarding the complex transcriptional mechanisms of the gigantic *DMD *gene.

## Methods

### Patient selection

A sample of 41 DMD and 6 BMD patients was shown to be negative for both deletions and duplications in the entire coding region of the *DMD *gene, as well as the adjacent splice site and muscle promoter regions, by multiplex ligation-dependent probe amplification (MLPA) and multiplex polymerase chain reaction (PCR) analysis.

Written informed consent was obtained (and preserved in original) from all subjects or their caregivers at the time of primary diagnostic procedures, with explicit consent to future uses for research purpose, according to the Declaration of Helsinki. This protocol was approved by the Institutional Review Board of the Fondazione I.R.C.C.S. Ca' Granda Ospedale Maggiore Policlinico - University of Milan.

The "Telethon Bank of DNA, Nerve and Muscle Tissues" (no. GTF02008), located in the Department of Neurological Sciences, Fondazione I.R.C.C.S. Ca' Granda Ospedale Maggiore Policlinico, Milan, Italy, was the source of the DNA samples used in this study.

Thirty-five probands showed a DMD clinical phenotype and the absence of dystrophin protein in muscle immunohistochemistry (IHC) and Western blot (WB) analysis with multiple anti-dystrophin antibodies, while six had a milder muscle involvement and variable muscle dystrophin expression consistent with a diagnosis of BMD.

In this cohort, we undertook a systematic study of sequence variation, by direct sequence analysis. The patients were regularly followed, with a complete clinical evaluation comprising cardiac, respiratory, and cognitive assessments.

### Muscle tissue analysis

Muscle biopsy was taken from brachial biceps or quadriceps muscle after written informed consent as previously reported. According to standard techniques, the muscle sections were analyzed by morphological study and IHC analysis. Dystrophin IHC was performed [21] using monoclonal antibodies against mid-rod domain, NH_2_, and COOH epitopes (Novocastra, Newcastle upon Tyne, UK), and was completed using α-SG, β-SG, γ-SG, δ-SG, dysferlin (Novocastra), and caveolin-3 (Transduction Laboratories, Lexington, KY) monoclonal antibodies (Santa Cruz Biotechnology, Santa Cruz, CA) as described [22-24]. Dystrophin defects revealed by IHC were confirmed by WB analysis using the same monoclonal antibodies.

### Molecular analysis

DNA was extracted from peripheral blood samples according to standard procedures (Flexi Gene DNA Handbook, Quiagen). All patients were screened for deletions and duplications with multiplex PCR and MLPA as previously described [25].

### Sequence analysis

All 79 exons and the exon-intron boundaries of the DMD gene were studied through PCR amplification and direct sequencing (ABI Prism 3100 Genetic Analyzer, Applied Biosystem, Foster City, USA) using published primer sets [26]. Mutations were named according to the Leiden Muscular Dystrophy database http://www.dmd.nl/ using the nomenclature system published in 2000 on Human Mutation [27] (reference sequence GenBank file NM_004006.1)

### Transcript analysis

Patients with mutations leading to abnormal mRNA splicing were further investigated through transcript analysis. In these patients, mRNA was isolated from muscle tissue with Eurozol (EMR055100, EuroClone). The cDNA was produced through reverse transcription polymerase chain reaction (RT-PCR) (Ready-To-Go RT-PCR kit, Amersham Pharmacia) and analyzed by amplification and sequencing. Transcript analysis was performed using primers previously published by Roberts et al. [26]. New primers were designed in some specific cases. All primer sequences are available on request.

### Bioinformatics analysis

To estimate the effect of the T/G mutation in intron 65 of the *DMD *gene, we used the splice site models introduced by Yeo and Burge [28] and the software available at: http://genes.mit.edu/burgelab/maxent/Xmaxentscan_scoreseq_acc.html. The sequences CTGGTAATT and CTGGTAAGT, which correspond respectively to the wild-type and mutated 5' splice site of the included exon were scored. We obtained maxENT scores, which represents the probability that a sequence is a functional splice site. Therefore, as suggested by Enl et al. [29], given two sequences of differing scores, the higher-scoring sequence has a higher likelihood of being used as 5' splice site.

## Results

We selected 47 patients (41 probands) carrying nucleotide alterations in the *DMD *gene; 41 patients were affected with DMD and 6 with BMD.

The distribution of the mutations in DMD families was as follows: 9 micro-insertions or microdeletions, 19 nonsense substitutions, 7 splice-site mutations.

**Figure 1:**
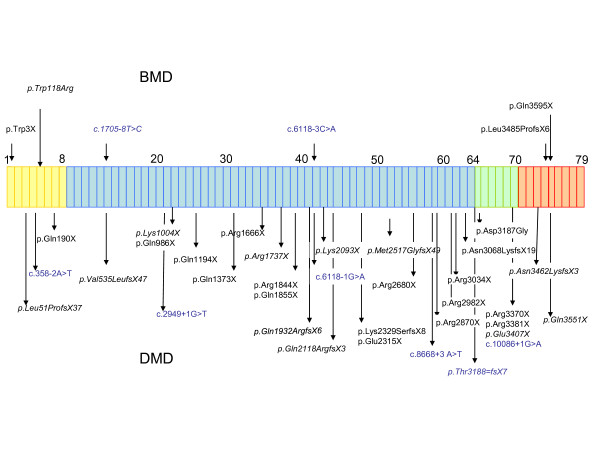
**Distribution of mutations along the *DMD *gene**. Each box corresponds to an exon, with different colours indicating the four different functional portions of the gene: actin-binding domain (exons 1-8, yellow); rod domain (exons 9-62, blue); cysteine-rich domain (exons 63-69, green); C-terminal domain (exons 70-79, red). Mutations identified in the BMD population are listed in the upper part of the figure, while mutations found in the DMD population are listed in the lower section. Intronic mutations are listed in blue.

Among BMD subjects, 2 carried nonsense mutations, 2 showed splice-site mutations, 1 had a missense substitution, and 1 patient presented with the insertion of a single nucleotide (Figure 1). The most frequent stop codon was TGA (n = 10 patients) followed by TAG (n = 7 patients) and TAA (n = 4 patients). Altogether, 16 mutations were novel.

The mean age at diagnosis of the entire DMD group was 2.8 ± 1.3 years (range 1-6 years) while BMD patients came to medical attention at approximately 14 ± 20 years (range 3-46 years). Fifty-six percent of DMD patients were wheelchair bound; they lost independent ambulation at a mean age of 10.1 years. The clinical and molecular characteristics of this population are summarized in Table 1.

**Table 1:** Clinical and molecular characteristics of patients carrying point mutations in DMD gene

**Pt N.**	**BMD vs DMD**	**Mutation**		**Effect of mutation**	**Exon/intron**	**Stop codon**	**Reference**	**Age of onset/diagnosis(ys)**	**Symptoms at onset**	**CK levels (UI/L)**	**Age CK**	**Loss of ambulation (age)**
*I*	*BMD*	*Insertion*	*c.10453insC*	*p.Leu3485ProfsX6*	*74*		[33]	*7*	*HyperCKemia*	*12922*	*7*	*No (9)*
II	BMD	Nonsense	c.9G>A	p.Trp3X	1	TGA	[10]	3	HyperCKemia	3300	3	No (7)
*III*	*BMD*	*Nonsense*	*c.10783C>T*	*p.Gln3595X*	*75*	*TAG*	[35]	*nd*	*nd*	*nd*	*nd*	*Yes (22)*
IV	BMD	Missense	c.352T>A	p.Trp118Arg	5		**Novel**	45	Motor impairment	nd	nd	No (49)
V	BMD	Splicing	c.1705-8T>C	cDNA analysis NOT done	IVS14		**Novel**	2	hyperCKemia	1604	2	No (5)
*VI*	*BMD*	*Splicing*	*c.6118-3C>A*	*cDNA analysis NOT done*	*IVS42*		***Novel***	*49*	*Motor impairment*	*1400*	*46*	*No (68)*
VII	DMD	Insertion	c.151insC	p.Leu51ProfsX37	3		**Novel**	nd	nd	nd	nd	nd
VIII	DMD	Duplication	c.3009dupTG	p.Lys1004X	23		**Novel**	nd	nd	nd	nd	nd
IX	DMD	Duplication	c.5773_5794dup	p.Gln1932ArgfsX6	41		**Novel**	1	HyperCKemia	2956	17	Yes (12)
X.1	DMD	Insertion	c.7548insGGCAACA	p.Met2517GlyfsX49	52		**Novel**	nd	nd	nd	nd	nd
X.2	DMD	Insertion	c.7548insGGCAACA	p.Met2517GlyfsX49	52		**Novel**	nd	nd	nd	nd	nd
XI	DMD	Deletion	c.6353delA	p.Gln2118ArgfsX3	44		**Novel**	2	HyperCKemia	16159	2	No (13)
XII.1	DMD	Deletion	c.6986delA	p.Lys2329SerfsX8	48		[37]	1	HyperCKemia	11493	1	No (1)
XII.2	DMD	Deletion	c.6986delA	p.Lys2329SerfsX8	48		[37]	3	Motor impairment	32359	3	No (4)
XIII	DMD	Deletion	c.9204_9207delCAAA	p.Asn3068LysfsX19	62		Leiden database	4	HyperCKemia	7671	6	Yes (8)
XIV	DMD	Deletion	c.10386delT	p.Asn3462LysfsX3	73		**Novel**	nd	nd	4760	11	Yes (12)
XV.1	DMD	Del/ins	c.1603delGTAinsCT	p.Val535LeufsX47	14		**Novel**	nd	nd	12000	9	Yes (13)
XV.2	DMD	Del/ins	c.1603delGTAinsCT	p.Val535LeufsX47	14		**Novel**	nd	nd	2061	13	Yes (12)
XV.3	DMD	Del/ins	c.1603delGTAinsCT	p.Val535LeufsX47	14		**Novel**	nd	nd	5357	9	Yes (10)
XVI	DMD	Nonsense	c.568C>T	p.Gln190X	7	TAG	[10]	3	Motor impairment	12800	6	No (6)
XVII	DMD	Nonsense	c.2956C>T	p.Gln986X	23	TAA	[35]	3	Motor impairment	10430	3	Yes (11)
XVIII	DMD	Nonsense	c.3580C>T	p.Gln1194X	26	TAG	[10]	3	HyperCKemia	13580	3	No (7)
XIX	DMD	Nonsense	c.4117C>T	p.Gln1373X	30	TAG	Leiden database	2	HyperCKemia	7246	5	No (6)
XX	DMD	Nonsense	c.4996C>T	p.Arg1666X	35	TGA	38]	1	Delayed motor milestones	8000	1	No (7)
XXI	DMD	Nonsense	c.5209C>T	p.Arg1737X	37	TAA	**Novel**	2	HyperCKemia	nd	nd	No (2)
XXII	DMD	Nonsense	c.5530C>T	p.Arg1844X	39	TGA	[38]	3	Motor impairment	19000	5	No (6)
XXIII.1	DMD	Nonsense	c.5563C>T	p.Gln1855X	39	TAG	[35]	3	HyperCKemia	19452	4	No (7)
XXIII.2	DMD	Nonsense	c.5563C>T	p.Gln1855X	39	TAG	[35]	2	No symptoms	nd		No (5)
XXIV	DMD	Nonsense	c.6277A>T	p.Lys2093X	43	TAG	**Novel**	1	Delayed motor milestones	17000	1	No (5)
XXV.1	DMD	Nonsense	c.6943G>T	p.Glu2315X	48	TAA	[39]	3	Motor impairment	1918	12	Yes (8)
XXV.2	DMD	Nonsense	c.6943G>T	p.Glu2315X	48	TAA	[39]	3	Motor impairment	7583	9	No (8)
XXVI	DMD	Nonsense	c.8038C>T	p.Arg2680X	55	TGA	[40]	4	Delayed speech	3211	11	Yes (9)
XXVII	DMD	Nonsense	c.8608C>T	p.Arg2870X	58	TGA	Leiden database	1	Delayed motor milestones	12954	9	No (10)
XXVIII	DMD	Nonsense	c.8944C>T	p.Arg2982X	60	TGA	[38]	4	Delayed motor milestones	8617	7	No (8)
XXIX	DMD	Nonsense	c.9100C>T	p.Arg3034X	61	TGA	[10]	6	Delayed motor milestones	15000	1	No (8)
XXX	DMD	Nonsense	c.10108C>T	p.Arg3370X	70	TGA	[40]	4	Delayed motor milestones	8891	1	No (9)
XXXI	DMD	Nonsense	c.10108 C>T	p.Arg3370X	70	TGA	[40]	4	Delayed motor milestones	2312	10	Yes (10)
XXXII	DMD	Nonsense	c.10141C>T	p.Arg3381X	70	TGA	[40]	nd	nd	nd	nd	No (4)
XXXIII	DMD	Nonsense	c.10219G>T	p.Glu3407X	70	TAA	**Novel**	4	HyperCKemia	9200	4	No (11)
XXXIV	DMD	Nonsense	c.10651C>T	p.Gln3551X	75	TAG	**Novel**	1	Delayed motor milestones	13672	2	nd
*XXXV*	*DMD*	*Splicing*	*c.9560A>G*	*p.Asp3187Gly*	*65*		[39]	*4*	*HyperCKemia*	*17634*	*5*	*No (6)*
XXXVI	DMD	Splicing	c.358-2A>T	cDNA analysis NOT done	IVS5		[10]	4	nd	3904	10	Yes (8)
XXXVII	DMD	Splicing	c.2949+1G>T	cDNA analysis NOT done	IVS22		[12]	nd	Delayed motor milestones	11834	1	nd
XXXVIII	DMD	Splicing	c.6118-1G>A	cDNA analysis NOT done	IVS42		Leiden database	nd	nd	nd		nd
XXXIX	DMD	Splicing	c.8668+3A>T	cDNA analysis NOT done	IVS58		**Novel**	3	HyperCKemia	11815	3	Yes (10)
*XL*	*DMD*	*Splicing*	*c.9563_9564ins53*	*p.Thr3188=fsX7*	*IVS65*		***Novel***	*4*	*HyperCKemia*	*16000*	*4*	*No (6)*
XLI	DMD	Splicing	c.10086+1G>A	cDNA analysis NOT done	IVS69		[36]	1	Delayed motor milestones	7365	1	Yes (9)

In the majority of patients, phenotype (DMD versus BMD) was predicted by genotype, according to the reading frame rule. However, we observed exceptions to this rule in 2 of 34 DMD cases and 4 of 6 BMD cases. Patient II, carrying the nonsense mutation p.Trp3X has been previously described (patient VII, R007) [30]. Clinical and molecular data regarding the other five cases, relevant to the issue of the reading frame rule or presenting rare aberrant mRNA maturation, are described in detail.

### Patient I: Creation of a stop codon at the 3' end of the DMD gene is associated with a mild BMD phenotype

A 7-year-old boy came to medical attention because of occasionally high creatine kinase (CK) levels (12,922 IU/L) during bladder infection. He was completely asymptomatic, showing only mild calf hypertrophy and Achilles tendon retraction, with no muscular weakness. He had neither cardiac nor respiratory impairment. Full Intelligence Quotient (IQ) level was normal.

**Figure 2:**
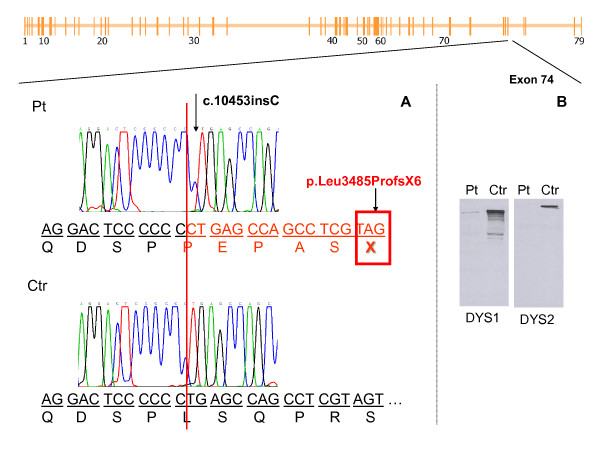
**Patient I: stop codon at the 3' end of *DMD *gene in mild BMD phenotype**. Molecular and muscle biopsy features in patient I. The patient showed a mild BMD phenotype and carried a mutation that creates a stop codon at the 3' end of the *DMD *gene. In the upper part of the figure, the schematic representation of the *DMD *gene depicts the position of the mutation, which is located in exon 74. A. Sequence analysis of genomic DNA. Pt (Patient I): The electropherogram depicts the single base pair insertion c.10453insC (black arrow). Under the electropherogram, the predicted amino acid sequence demonstrates the effect of the mutation, which leads to the out-of-frame transcript p.Leu3485ProfsX6. Ctr: Control. B. Dystrophin Western blot analysis of a muscle biopsy sample from patient I revealed decreased immunoblot staining with antibody directed against the dystrophin mid-rod domain (DYS1), and absence of signal with antibodies directed towards the C-terminal epitope (DYS2). Ctr: Control muscle sample.

A muscle biopsy performed on the brachial biceps at 7 years of age showed a mild dystrophic pattern with moderate connective tissue increase, fibre size variability, and 3-4 necrotic fibres. IHC revealed decreased reactivity with antibody against NH2 and Rod-domain and complete absence of signal with antibodies directed towards the COOH-terminal epitope. Molecular analysis demonstrated the presence of a single nucleotide insertion in exon 74 (c.10453insC). This insertion leads to a frame shift mutation with the creation of a premature stop codon (p.Leu3485ProfsX6) (Figure 2A) and does not fit with the mild clinical presentation of the patient.

The protein data were further confirmed by dystrophin WB analysis on muscle homogenate, which showed the presence of a truncated protein lacking of the C-terminal domain (Figure 2B); although decreased in molecular weight and amount, this protein is still able to localize to the membrane as demonstrated by IHC.

### Patient III: Nonsense mutation located at the 3' end of the DMD gene is associated with a severe BMD phenotype

This patient presented with a severe BMD phenotype. He started to complain muscular impairment with difficulties in running and climbing stairs at 9 years of age. Motor involvement displayed a fast, progressive course with loss of independent ambulation at 22 years of age. At 19 years of age, he also developed severe cardiac involvement with arrhythmia and hypocinesia. After 3 years, he presented with a reduction of ejection fraction (40%) and started cardiac therapy. He also presented with a restrictive respiratory pattern, but did not require non-invasive mechanical ventilation. At 27 years of age, this patient had stable clinical parameters.

**Figure 3:**
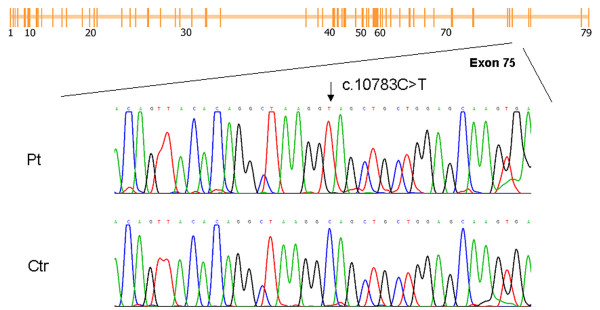
**Patient III: Nonsense mutation at the 3' end of *DMD *gene in severe BMD phenotype**. The mutation is localized in exon 75 (see schematic representation of the *DMD *gene).. Sequence analysis revealed the nucleotide substitution c.10783C>T (black arrow). This mutation leads to the creation of a stop codon (p.Gln3595X).

Muscle tissue analysis revealed a severe dystrophic pattern and weak sarcolemmal signal with DYS rod-domain antibodies. After exclusion of deletions and duplications of the *DMD *gene, sequence analysis showed a single nucleotide substitution (c.10783C>T) (Figure 3) that is predicted to create a stop codon in exon 75 (p.Gln3595X). This "null" mutation was also compatible with partial dystrophin production, even if the quantity of protein, as evaluated by WB analysis, was lower than in the case of Patient I.

### Patient VI: Leaky splice site mutation and double transcript in a BMD patient

The patient showed a BMD phenotype with late onset, at 49 years of age. He complained of pain, muscle wasting of the quadriceps, and lower limb girdle muscle weakness. Although the disease had a progressive course, the patient was still ambulant at 68 years of age, manifesting only mild difficulty in postural adjustments.

A muscle biopsy conducted at 50 years of age revealed mild myopathic changes with a slight lipid increase. A repeated biopsy performed at 58 years of age demonstrated a dystrophic pattern with fibre size variability, as well as several nuclear centralizations and fiber splittings. Connective tissue was normal in all samples. IHC analysis revealed mild sarcolemmal staining with the three antibodies. WB showed a mild intensity reduction with both DYS1 and DYS2 antibodies, and sarcoglycan immunostaining was also decreased. The screening of the *DMD *gene for deletions and duplications with multiple-PCR and MLPA and the analysis of the four sarcoglycan genes by direct sequencing showed no mutations.

**Figure 4:**
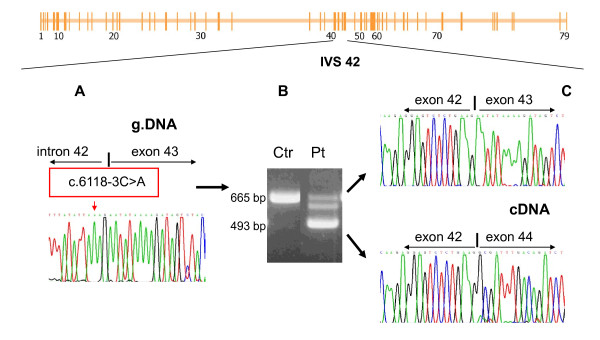
**Patient VI: Leaky splice site mutation and double transcript in a BMD patient**. The mutation is localized within intron 42 (see schematic representation of the *DMD *gene reproduced in the upper part of the figure). A. Genomic analysis of the 3' splice site of intron 42 in patient VI. The electropherogram depicts the nucleotide substitution c.6118-3C>A in IVS42 (red box). B. Agarose gel electrophoretic analysis of PCR products derived from the RT-PCR of *DMD *cDNA nucleotides 5959-6624 form patient VI's muscle-derived cDNA. Transcript analysis reveals the presence of two different RT-PCR products in the patient (Pt): the expected 665-bp full-length band, and a shorter 493-bp band; the RT-PCR product from a control subject demonstrates the presence of the wild-type 665-bp band. C. Sequence analysis of the RT-PCR products demonstrates that the 665-bp band corresponds to the full-length cDNA, while the 493-bp band corresponds to a product carrying the deletion of exon 43.

*DMD *gene sequencing revealed a c.6118-3C>A nucleotide substitution in IVS42 (Figure 4A). This novel mutation is expected to determine the skipping of exon 42 with production of an out-of-frame transcript associated with severe DMD phenotype.

The mild clinical presentation of Patient VI was explained by transcript analysis.

In fact, study of the transcript revealed the presence of two different products (Figure 4B): a 665-bp band corresponding to full-length PCR products (Figure 4B, upper transcript) and a more intense band of lower molecular weight, corresponding to the exon 43-deleted product (Figure 4B, lower transcript). The data were therefore consistent with the hypothesis that a leaky out-of-frame splice defect led to the production of a decreased amount of normal full-length DYS cDNA and a decreased amount of normal dystrophin.

### Patient XXXV: Coding region point mutation activates a cryptic exonic splice site

A 4-year-old boy presented with occasionally high CK levels (17,634 IU/L) confirmed at further analysis. At 6 years of age, he displayed moderate proximal muscle weakness in the upper and lower limbs with positive Gowers' manoeuvre, and was started on corticosteroid therapy. Patient XXXV also showed mild cognitive impairment with difficulties at elementary school. He had no cardiac or respiratory involvement.

**Figure 5:**
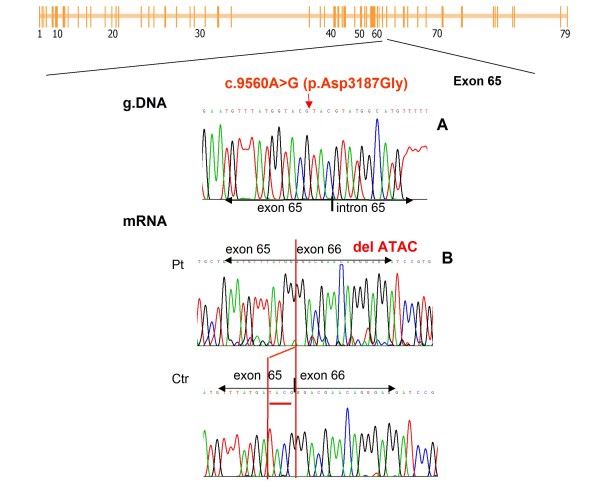
**Patient XXXV: Coding region point mutation activates a cryptic exonic splice site**. The mutation is localized in exon 65 (see schematic representation of the *DMD *gene). A. Sequence analysis. An electropherogram depicting the nucleotide substitution c.9560A>G, which would putatively result in an amino acid substitution (p.Asp3187Gly). B. Transcript analysis. The nucleotide substitution activates a cryptic splice site with skipping of the last 4 nucleotides of exon 65 (Pt: patient; Ctr: control), which changes the reading frame.

Muscle biopsy obtained from brachial biceps demonstrated a dystrophic pattern. IHC analysis showed a complete absence of dystrophin. Multiplex-PCR and MLPA analysis revealed no deletions or duplications. Gene sequencing revealed a novel substitution (c.9560A>G) in one of the last nucleotides of exon 65 (Figure 5A). Although the mutation predictably causes a missense mutation that results in an amino acid change at position 3187 (p.Asp3187Gly), this hypothesis did not fit with the clinical presentation of the patient.

Through transcript analysis we ascertained that this nucleotide substitution determines the activation of a cryptic splice site, resulting in skipping of the last four nucleotides of exon 65 with consequent change of the reading frame and creation of an out-of-frame transcript (Figure 5B). Nucleotide substitutions located near intron-exon boundaries mimicking missense mutations can modify the splicing process, creating a new splice site even if they are not located in typical splice sites.

### Patient XL: An intronic nucleotide substitution activates a cryptic splice site and leads to pseudoexon creation

This patient came to our attention as a 4-year-old boy presenting with occasionally high CK levels (7,000 IU/L). He was the first boy of healthy parents with a family history negative for muscle disorders. At first evaluation, he showed mild calf hypertrophy and frequent falling. During the following two years he developed mild proximal lower limb weakness with Gowers' sign and running difficulties. Patient XL never learned to run and was able to climb the stairs only with the aid of a handrail. He had no cardiac, respiratory, or cognitive impairments.

At 6 years of age, Patient XL underwent muscle biopsy on the brachial biceps. Muscle tissue analysis revealed a dystrophic pattern with moderate fibre size variability, rare nuclear centralization, and fibre splittings, as well as few instances of necrosis. Interstitial cellular infiltrates were absent. Connective tissue was increased. IHC analysis demonstrated the complete absence of dystrophin on muscle membrane with the antibodies directed against all three epitopes. A mild reduction with α-SG antibodies was also present.

**Figure 6:**
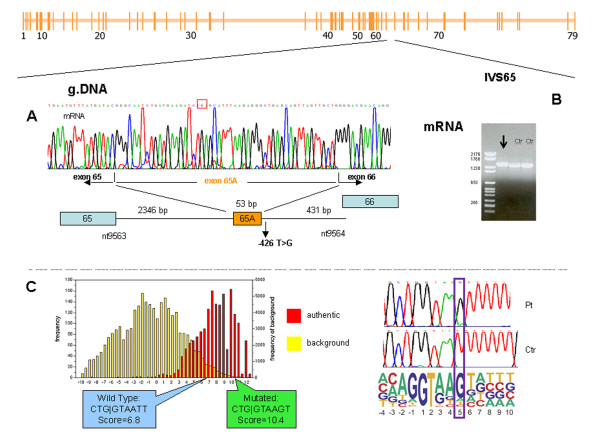
**Patient XL: An intronic nucleotide substitution activates a cryptic splice site leading to pseudoexon creation**. The mutation is localized in intron 65 (see schematic representation of the *DMD *gene). A. Sequence analysis. The electropherogram depicts the insertion of 53 bp between exons 65 and 66. This insertion creates a stop codon (TGA, red box) and results in the production of a truncated protein (p.Thr3188ThrfsX7). The insertion is determined by a nucleotide substitution (c.9564-426T>G) in IVS 65, which activates a cryptic splice site. The schematic representation of IVS65 under the electropherogram depicts the position of the intronic mutation and the sequence inserted into intron 65. B. cDNA analysis depicts a band with higher molecular weight than control (Ctr) in the patient, demonstrating the insertion of 53 bp. C. Bioinformatic analysis: 5' Splice Site score distribution in patient (mutated, green) versus control (wild type, blue). The mutation is localized in a silent splice site consensus sequence included in the intron and increases the MaxENT score, that gives the probability that a sequence is a functional splice site, from a value of 6.8 to a value of 10.4. The obtained maxENT scores, suggest that the mutation activates a cripitc 5'ss leading to inclusion of the exon in the mature transcript. The substitution involves one of the most important and conserved bases of the consensus sequence, as shown in the schematic representation on the right, which illustrates the relative frequency of different bases in the 5' splice site consensus sequence (the size of the letter indicating each nucleotide reflects its importance in the consensus sequence).

The *DMD *gene was investigated, with multiple-PCR and MLPA excluding the presence of deletions and duplications. Sequence analysis did not demonstrate the presence of point mutations. cDNA analysis showed the existence of a band of higher molecular weight than expected (Figure 6A). Direct sequencing of RT-PCR products revealed the insertion of 53 bp (c.9563_9564ins53) between exons 65 and 66 (Figure 6B). Further analysis demonstrated that the inserted fragment corresponded to a portion of intron 65. Its insertion into natural transcript determines the creation of a premature stop codon (p.Thr3188 = fsX7), which is responsible for the complete absence of dystrophin at IHC analysis.

To better understand the molecular mechanism underlying this genetic rearrangement, we further investigated genomic sequence and found a nucleotide substitution at position -426 in intron 65 (c.9564-426 T>G); this mutation is located 5 bp downstream from the inserted sequence. It has not been previously described, and had a "de novo" origin since it was not present in the patient's mother.

Bio-informatic analysis (Figure 6C) showed that this mutation is localized in a silent splice site consensus sequence included in the intron. The substitution involves one of the most important and conserved bases of the sequence and markedly increases the splice site strength, changing the maxENT score from a value of 6.8 in wild type to a value of 10.4 in the patient, and making it higher than is found in normal exons (8.1). The activation of this criptic 5'splice site and its interaction with one pre-existing, but normally silent, acceptor splice site within intron 65 leads to the insertion of a 53-bp pseudoexon.

## Discussion

We performed a clinical and molecular study of a sample of 47 Italian dystrophinopathic patients (41 DMD and 6 BMD) carrying nucleotide alterations in the *DMD *gene. All types of mutations were represented, including 10 micro-insertions or deletions, 21 nonsense substitutions, 9 splice-site mutations, and 1 missense mutation. Overall 16 of these mutations had not been previously described.

From this cohort, we presented in detail five cases paradigmatic of mechanisms that are responsible for complex rearrangements or novel exceptions to the reading frame rule. Two patients carried nonsense mutations associated to unexpected BMD phenotype, the other three represent unconventional splice-site mutations, not localized in canonical splice sites or with variable transcriptional effects. Three of these five interesting mutations are also novel.

Nonsense mutations potentially determine the interruption of dystrophin synthesis, mRNA decay, and degradation of truncated dystrophin molecules, unless they are located in the 5' or 3' gene regions [31,32]. In particular, two mechanisms are thought to be responsible for the variable effect of nonsense mutations located in these gene regions, namely initiation of translation from downstream ATG codons (in the case of mutations located at the 5' end) and escape from nonsense-mediated decay (for substitutions in the 3' portion). In our sample, patients I and III carried a nonsense mutation located at the 3' of the gene associated with a BMD phenotype of variable severity, with partial production of dystrophin protein demonstrated by IHC and WB analysis (Figure 2). Similar results have been described in other cases. A detailed analysis of the Leiden database http://www.dmd.nl indicates that the majority of mutations located in the final 5 exons of the dystrophin gene, even if determining production of an out-of frame transcript, are associated with BMD or intermediate phenotypes; only 11 are associated with DMD. In particular, the c.10453insC mutation has been described in 5 different patients: 1 showed a DMD phenotype; 2 had a mild Becker-like muscular involvement; and 2 subjects, of African and French origin, presented with an intermediate phenotype [33-34, Leiden muscular dystrophy database].

The mutation c.10783C>T has been described once [35] in one DMD patient. We hypothesize that the mutations located at the end of DMD cDNA bypass mRNA decay as previously demonstrated [32], preserving the protein functional domains responsible for anchorage of the protein to the membrane.

While the efficiency of nonsense-mediated mRNA decay can differ among patients carrying different distal mutations, leading to different phenotypes, the mechanisms influencing this process are not known. Furthermore, this high spectrum of variability, which is also evidenced among BMD presentations, suggests the influence of other factors that remain to be investigated. The exact location of the mutation may also be considered an important factor. It is worth mentioning that in some cases nonsense mutations at the 3' end of the gene can be associated with the DMD phenotype, as well, as we observed in two patients carrying two nonsense mutations in exons 74 and 75 (p.Gln3551X; p.Asn3462LysfsX3).

Interesting insights into dystrophin mRNA maturation may be derived from patients VI, XXXV, and XL, who carry different mutations affecting the splicing process.

The mechanisms that determine alternative splicing are complex and still not completely characterized. To date, disease-causing splicing mutations described in DMD and BMD populations seems to mainly affect canonical splice site sequences; the effect of these mutations on mRNA processing has been investigated in few published cases [17].

The behaviour of mutations involving the splice site is not always completely predictable: in patient VI a leaky novel splice site mutation determines the simultaneous creation of two PCR products reflecting the presence of both a full length and a spliced transcript (Figure 4).

The efficiency of the novel splicing signal inversely correlates with disease severity and must be considered when predicting the effect of the mutation. The case of patient XXXV demonstrates how splicing can be altered not only by mutations localized in the canonical splice sites, but also by mutations in the neighbouring exonic sequences. A nucleotide substitution located near the exon-intron boundary, first interpreted as a missense mutation, was demonstrated to have a strong effect on splicing. This substitution determines the activation of a cryptic splice site, resulting in skipping of the last four nucleotides of exon 65, with a consequent reading frame shift resulting in a severe phenotype (Figure 5).

Finally, splicing can also be altered by mutations of deep intronic regions located at sites that can be recognized as splice site consensus sequences. In these cases, transcript analysis associated with bioinformatic predictions may allow the clarification of molecular mechanisms underlying the disease phenotype. So far, the creation of additional splice sites in the central part of an intron has been reported in few cases [13,36]. In patient XL, a single nucleotide substitution in a consensus sequence that is normally silent increases its efficiency and determines the insertion of a pseudo-exon (Figure 6).

## Conclusion

All these cases underscore the importance of a complete study of the gene, including exon-intron boundary sequencing and transcript analysis, which has been demonstrated to be the only definitive analysis. Nonetheless, muscle biopsy remains highly informative because dystrophin mRNA is hardly extracted from blood samples and dystrophin protein can be analyzed in muscle tissue.

Also, when a complete molecular characterization is achieved, the interpretation of genetic data "per se" might be challenging. The reading frame rule can be used to predict the effect of the majority of mutations, but some exceptions have been described, especially among nucleotide alterations. Occasionally, these exceptions may provide support for another general rule, as it occurs with the stop codon point mutations located in the 5' part of the DMD gene whereby the initiation of translation occurs from downstream AUG codons and results in BMD phenotypes [30]. However such a general rule can be hardly recognized for patients carrying nonsense mutations located at 3' part of the gene, that may lead to DMD, intermediate or BMD phenotypes without an apparent rationale. In other instances, such as the splicing variants described in patient VI and XXXV, the molecular data evaluation should incorporate both mRNA data and bioinformatic predictions in order to draw a reliable conclusion on expected phenotype.

These instances support the notion that only a thorough investigation of clinical features, muscle biopsy data and molecular findings would finally give to the molecular data interpretation a prognostic value in all dystrophinopatic patients.

## Competing interests

The authors declare that they have no competing interests.

## Authors' contributions

FM and AG contributed to conception and design, acquisition and analysis of data, and drafted the manuscript. RB and SG carried out the molecular genetic studies. AB and ST performed respectively the DMD gene study through Multiplex-PCR analysis and MLPA. PC, FF, VL performed muscle tissue analysis and carried out the immunoassays. MGD and SG contributed in patients' selection and follow-up and conducted the clinical examination and phenotyping. SC, MM have been involved in acquisition and analysis of data and manuscript drafting, revising it critically for important intellectual content. NB participated in the design of the study. MC performed bioinformatics analysis. GC supervised all the work, conceived of the study, participated in its design and coordination, and helped to draft the manuscript; finally he gave final approval of the version to be published. All authors read and approved the final manuscript.

## Pre-publication history

The pre-publication history for this paper can be accessed here:

http://www.biomedcentral.com/1471-2350/12/37/prepub
